# Association of Vitamin A Status with Overnutrition in Children and Adolescents

**DOI:** 10.3390/ijerph121214998

**Published:** 2015-12-07

**Authors:** Chun Yang, Jing Chen, Zhen Liu, Chunfeng Yun, Yajie Li, Jianhua Piao, Xiaoguang Yang

**Affiliations:** Key Laboratory of Trace Element Nutrition, National Health and Family Planning Commission of China, Department of Trace Element Nutrition, National Institute for Nutrition and Health, Chinese Center for Disease Control and Prevention, Beijing 100050, China; inyingyangchun@163.com (C.Y.); chenjing1909@sina.com (J.C.); janet604@126.com (Z.L.); sallyycf@163.com (C.Y.); yjsome@163.com (Y.L.); piaojh@163.com (J.P.)

**Keywords:** vitamin A, body mass index, children, adolescents

## Abstract

This study was conducted to examine the possible association between vitamin A status and overnutrition in Chinese urban children and adolescents. Weight, height and serum retinol were assessed in total 3457 children (7–9.9 years of age) and adolescents (10–17 years of age), using urban region data from the China National Nutrition and Health Survey 2010–2013 (CHNNS2010-2013) which is a nationally representative cross-sectional study. Prevalence of low serum concentration of retinol was 26.8% and 12.24% for overweight. Retinol inadequacy was significantly higher in children (32.13%) than in adolescents (24.48%). The average of retinol was significantly higher in overnutrified 42.32 μg/dL *versus* non-overnutrified 41.05 μg/dL (*p* = 0.00) children and adolescents. Overnutrified children and adolescents presented a greater chance of an increase in serum concentration of retinol (odds ratio 1.34, 95% confidence interval 1.10–1.63, and 1.48, 95% confidence interval (1.26–1.74) when compared with non-overnutrified children. An important correspondence between vitamin A deficiency and overnutrition was found. Non-overnutrified children and adolescents may have a greater chance of presenting low concentrations of retinol. Future public health strategies focused on the overnutrified population and vitamin A supplements should consider the effect of retinol on urban children and adolescents in China.

## 1. Introduction

Vitamin A is an essential nutrient needed in small amounts for the normal functioning of the visual system, and maintenance of cell function for growth, epithelial integrity, red blood cell production, immunity and reproduction [1]. The World Health Organization (WHO) recommends measuring serum retinol concentrations as the major approach to assess vitamin A status in a population [1]. Vitamin A deficiency (VAD) is a tissue concentration of vitamin A low enough to have adverse health consequences, even if there is no evidence of clinical deficiency [2]. VAD is recognized as a major public-health nutrition issue in developing countries [3]. VAD is more damaging to and predominantly affects preschool children, pregnant women and nursing mothers [1]. Overnutrition (overweight and obesity) has become a serious public health issue that affects a large proportion of the population worldwide among all age, gender and racial/ethnic groups [4]. Overnutrition in the pediatric population needs to receive careful attention because childhood and adolescence are key developmental periods during which individuals set up the foundations for their future health. Moreover, overnutrition among children and adolescents is associated with obesity and cardiovascular diseases in their adulthood [5]. Overnutrition in children and adolescents has been linked to medical health problems and poorer neurocognitive functioning, such as executive functioning, attention, visuo-spatial skills, and motor skills [6]. It is also a risk factor for diabetes, arterial hypertension, coronary artery disease, and fatty liver disease [7].

The weight increase among children and adolescents has been changing in the Chinese population, along with China’s dramatic economic and nutritional transition [8]. China now faces a significant and rapidly growing incidence of overnutrition in children and adolescents [9]. Among the Chinese pediatric population, the prevalence of overweight among children aged 6 to 18 years rose from 6.3% in 1991 to 8.8% in 2000 and to 17.1% in 2011 [10]. Vitamin A plays important role in the growth and physical development of children and adolescents. Children 7 to 17 years old are characterized by rapid growth, development of secondary sexual characteristics, and reproductive capacity, phenomena that need vitamin A participation. Some studies have shown that normal-weight adults and children have higher blood concentrations of vitamins and minerals compared with overweight and obese individuals [11,12], while other studies have observed opposite results [13,14]. In recent years, studies have found that vitamin A has an impact on the expression of lipids, inflammation and insulin in humans and obese animal models [13,15]. A 2011 study found that compared with children from rural areas, children from urban areas in China had a higher prevalence of overnutrition [16]. Data about vitamin A levels in Chinese urban children and adolescents are rare. Meanwhile little is known about the relationship between vitamin A and overnutrition in Chinese urban children and adolescents. Therefore, the present study aimed to assess vitamin A status and examine the association with overnutrition of 7 to 17 year old children and adolescents in Chinese urban areas using data from the China National Nutrition and Health Survey 2010–2013.

## 2. Materials and Methods

### 2.1. Study Design and Participants

Data for the present analysis were obtained from the China National Nutrition and Health Survey 2010–2013 (CHNNS2010–2013) [17]. This survey is a nationally representative cross-sectional study conducted by the Chinese Center for Disease Control and Prevention to assess the health and nutrition of Chinese civilians. It covered all 31 provinces, autonomous regions, and municipalities directly under the central government throughout China (except for Taiwan, Hong Kong, and Macao). A stratified multistage probability sampling design was used for the selection of participants. The country was divided into four strata by their economic characteristics and social development. These were large cities, small to medium cities, general rural areas and poor rural areas. It involved the random selection of 150 monitoring sites representing districts (urban) or counties (rural) of national coverage. Our study chose children in urban areas, which involved 34 monitoring sites distributed in large cities and 41 monitoring sites in small to medium cities, respectively. Each site chose the 7 to 17 year children and according to age and sex randomly selected five children. The technical problems of sampling blood and incomplete data were taken into consideration, so the final sample was formed by 3457 participants whose serum concentrations of retinols were available in the data bank.

### 2.2. Ethical Considerations

The study was conducted according to the guidelines laid down in the Declaration of Helsinki and all procedures involving human subjects were ethically approved by the Ethics Committee of the Institute for Nutrition and Health, Chinese Center for Disease Control and Prevention with the file number of 2013-018. All participants in this survey signed an informed consent form.

### 2.3. Other Relevant Variables

Children’s sex and age were collected through the use of a general information questionnaire with the help of mother or a mother substitute. Anthropometrical measurements were conducted by well-trained health workers who followed a reference protocol recommended by the WHO [18]. Body mass index (BMI) was calculated using height and weight measures (BMI = weight divided by height squared). Criteria adapted from the WHO guidelines were used to classify the nutritional status as follows: underweight was defined as below the 5th percentile [18,19]. Considering the differences in body composition across different ethnic groups, the Working Group for Obesity in China organized by the International Life Science Institute Focal Point in China conducted an analysis of BMI of children and adolescents aged 7–18 years. A new BMI classification reference was recommended by the Working Group for Obesity in China in 2004. Normal weight as equal to or above the 5th percentile and below the 85th percentile, overweight as equal to or above the 85th percentile and below the 95th percentile, and obese as equal to or above the 95th percentile [20], relative to sex and age. This standard is the most appropriate one and has been applied extensively in recent years.

To examine the association between overnutrition and serum concentrations of retinol, subjects classified as overweight or obese were grouped together in the overnutrition group and compared with those in the non-overnutrition group (eutrophic and underweight combined) because, when the analyses were carried out by categories, no significant differences in serum concentrations of retinol were found between overweight and obese and between eutrophic and underweight subjects.

### 2.4. Blood Sample Collection, Serum Extraction, and Laboratory Analysis

Fasting blood samples were obtained by venipuncture from the antecubital vein in the morning of the next day after questioning, and centrifuged within 0.5–1 h of collection. Serum was extracted and aliquoted immediately into screw-top vials. The samples were transported in iceboxes, protected from direct light, and stored at −80 °C until analyzed.

Serum retinol concentrations were analyzed using high performance liquid chromatography [21]. Retinol was extracted with hexane after deproteinization with ethanol containing retinyl acetate as the internal standard and evaporated to dryness with nitrogen gas. The residual materials were dissolved in 0.2 mL ethanol. The mobile phase was a methanol: DH_2_O mixture (96:4). A portion (10 μL) of the sample was injected into a column (3.9 × 150 mm; Symmetry Shield RPl8 Waters Breeze Milford, MA, USA) installed in a high-performance liquid chromatographic apparatus (Waters 1525 Binary HPLC Pump, Waters Breeze). All procedures were performed in a dark room to protect the serum from light. The concentration of retinol was determined with a spectrophotometer (Waters 2487 Dual Absorbance Detector, Waters Breeze) at 325 nm. Duplicate analyses were performed on one-tenth of the samples and the estimated variability was 0.02 μmol/L. The experienced examiners measured all biochemical indices. The inadequate of retinol was cutoff values below 30 μg/dL [22] previously reported in Brazil for children of the same age group [12] and based the evidence linking subclinical or prepathologic lack of vitamin A to increased morbidity/mortality risk [23,24].

### 2.5. Statistical Analysis

SAS version 9.2 (SAS Institute, Inc., Cary, NC, USA) was used for data-entry, screening, and analysis. Analyses were stratified by gender and age groups of 7 to 9.9 years (children) and 10 to 17 years (adolescents). Measurements of central tendency and dispersion for continuous variables were calculated. For comparison between the two averages, Kolmogorov-Smirnov test and unpaired Student’s t test was applied when Levene’s test for equality of variances was not significant. When it was significant, the modified t-test for different variances was used. Comparison of frequencies of categorical variables was done with the chi-square test. A multivariate analysis through the logistic regression model was used to test each independent variable associated (overnutrition, gender, and age range) with the dependent variable (low serum concentrations of retinol), using the backward method and then determining the logistic regression coefficients, odds ratios, and their 95% confidence intervals. The correlation between continuous variables was assessed with Pearson’s partial correlation coefficient. The established significance level was <0.05.

## 3. Results

The composition of the total sample was 3457 subjects, of which 51.20% were male (n = 1770), 48.80% female (n = 1687), 35.70% children (n = 1235), and 64.30% adolescents (n = 2404). The nutritional status across different age groups and gender was statistically significant (*p* = 0.03, and 0.00, respectively). The BMI averages were significantly lower for children than for adolescents (*p* = 0.00) and for males than for females (*p* = 0.00). The prevalence of retinol inadequacy was statistically higher for children when compared with adolescents. The average of retinol was statistically higher for adolescents when compared with children. No significant differences were observed between children and adolescents and between females and males in the other variables (Table 1).

Pearson’s correlation coefficient adjusted for age and gender was positive and significant (r = 0.06, *p* = 0.00) for BMI *versus* serum concentrations of retinol. Statistically significant differences were observed in the averages of retinol between overnutrified and non-overnutrified groups; the average of retinol was significantly higher (*p* = 0.00) for the overnutrition group (42.32 μg/dL) when compared with the non-overnutrition group (41.05 μg/dL; Table 2).

The logistic regression results (Table 3) showed a significant association with non-overnutrition and with age range. Compared with overnutrified children, non-overnutrified children had lower levels of retinol and were 1.34 times more likely to display inadequate serum concentrations of retinol. Compared with adolescents, children were 1.48 (1.26–1.74) times more likely to show inadequate serum concentrations of retinol. There was no significant association between inadequacy of retinol and gender.

## 4. Discussion

In the present study, 12.24% of children and adolescents were overweight, and 9.38% of children and 6.79% of adolescents were obese, which were both higher than the prevalence reported in previous studies [10]. We must highlight the importance of preventive measures in children and adolescents to reduce weight, especially in children. Overweight children were 2.8 times more likely than non-overweight children to become overweight adolescents [25]. Differences in overnutrition between males and females were found in the present study. Males were more likely to be overnutrified than females, which was consistent with the results from other Chinese studies [8,10,26]. Some studies in Western countries have also observed that sex differences in the prevalence of overnutrition were common among children and adolescents [27,28]. Males and females differ in body composition, patterns of weight gain, hormone biology, and the susceptibility to certain social, ethnic, genetic, and environmental factors, which contribute to the gender differences [29]. Thus, any proposed treatment and prevention of childhood overnutrition should account for these differences.

The rate of inadequate retinol values was 26.8% in the present study. Compared with other country studies of children in the same age group, inadequate retinol values of 38.8% were found in Brazil [30] and 0.9% in England [31]. There was higher prevalence of inadequate retinol values and lower levels of retinal were observed in the children in our study. The reason may be lower activity of child’s immune systems than in adolescents [32].

**Table 1:** Characteristics of children and adolescents according to age range and gender *.

Variables	Total	Children	Adolescents		*p*	Male	Female		*p*
Nutritional state									
Low weight	74 (2.14)	19 (1.80)	55 (2.29)	X^2^ = 8.75	0.03 ^†^	35 (1.98)	39 (2.31)	X^2^ = 67.64	0.00 ^†^
Eutrophic	2698 (78.04)	801 (75.92)	1897 (78.98)			1288 (72.77)	1410 (83.58)		
Overweight	423 (12.24)	136 (12.89)	287 (11.95)			278 (15.71)	145 (8.60)		
Obese	262 (7.58)	99 (9.39)	163 (6.78)			169 (9.54)	93 (5.51)		
Total	3457 (100)	1055 (100)	2402 (100)			1770 (100)	1687 (100)		
Serum concentrations of retinol									
Inadequate (<30 μg/dL)	927 (26.81)	339 (32.13)	588 (24.48)	X^2^ = 21. 88	0.00 ^†^	490 (27.68)	437 (25.90)	X^2^ = 1.39	0.24 ^†^
Adequate (≥30 μg/dL)	2530 (73.19)	716 (67.87)	1814 (75.52)			1280 (72.32)	1250 (74.10)		
Total	3457 (100)	1055 (100)	2402 (100)			1770 (100)	1687 (100)		
Body mass index (kg/m^2^)	18.69 ± 3.36	16.72 ± 2.47	19.55 ± 3.33	T = 27.87	0.00 ^§^	18.96 ± 3.54	18.40 ± 3.12	T = 4.97	0.00 ^§^
Serum concentrations of retinol (μg/dL)	41.30 ± 19.10	39.93 ± 20.79	41.91 ± 18.29	T = 2.81	0.01 ^‡^	41.23 ± 19.39	41.39 ± 18.80	T = −0.24	0.81 ^‡^

* Number (%) of subjects or average ± SD; ^†^ Chi-square test; ^§^ The t test modified for different variances; ^‡^ The *t* test.

**Table 2:** Averages, standard deviations, and 95% confidence intervals of serum concentrations of retinol in overnutrition and no-overnutrition children and adolescents.

Variables	n	Average	SD	95% CI	*p* ^‡^
Serum concentrations of retinol					
Non-overnutrition	2772	41.05	19.49	40.33–41.78	<0.05
Overnutrition	685	42.32	17.41	41.01–43.62	

^‡^ Z = 2.91, *p* = 0.00, Kolmogorov-Smirnov test.

**Table 3:** Results of logistic regression for association of non-overnutrition, gender, and age range to inadequacy serum concentrations of retinol.

Variables	β	P	OR (95% CI)
Serum concentrations of retinol			
Non-overnutrition	0.29	0.00	1.34 (1.10–1.63)
Age range	0.39	<0.00	1.48 (1.26–1.74)
Gender	-	0.11	-

CI, confidence interval; OR, odds ratio.

The low level of immunity can easily cause different infectious diseases [33,34,35,36]. A recent study found plasma retinol concentrations decreased because of infectious diseases in children [37]. Compared with adolescents, children were 1.48 (1.26–1.74) times more likely to have inadequate serum concentrations of retinol. These results are consistent with previous studies [30,38] demonstrating greater vulnerability of children. This tendency maybe be due to physical growth differences, the adverse effects of virus and bacterial infections, as well as parasitic infections to which children are prone, or even because of a greater dietary diversification chosen by adolescents [33,34,35,36,37]. Our results are of great relevance due to the fact the children population is considered an important population segment, and is a high risk group for vitamin A deficiency, so programs of prevention and correction of vitamin A deficiency are included in this period of life.

In this study, a positive correlation was also observed between serum concentrations of retinol and child’s BMI, for which similar results have been demonstrated in previous studies [13,14]. Our results were different from some studies [11,39,40], which found that low serum concentrations of retinol were associated with higher BMI. The different associations appeared on account of the fact those studies chose a population with higher BMI as subject, such as morbidly obese patients, children with high adiposity, who had abnormal adipose tissue biology in which lipophilic vitamins were sequestered in adipose tissue and this led to a decrease in serum micronutrient level [41]. Animal experiment studies also found that lower vitamin A status occurs in obese subjects, as the intestinal absorption of this vitamin is reduced by the increase of the synthesis of free fatty acids and triglycerides and by the inhibition of adaptable thermogenesis [42]. Obesity is defined as a positive balance between what is ingested and what is spent. The reasons causing obesity are directly related to the quantity and quality of the food which is consumed where there is a rich intake of calories and a poor intake of micronutrients [43]. The main etiologic factor for lower vitamin A at the epidemiologic level is the inadequate intake of dietary sources to satisfy the physiological needs of the individual [3]. The differences of relationship between retinol and BMI suggested the effect of retinol on adipose biology differs in individuals with BMI. In the present study, compared with overnutrified children, non-overnutrified children was lower levels of retinol and were 1.34 (1.10–1.63) times more likely to have inadequate serum concentrations of retinol. The reason may be that vitamin A, as a fat-soluble vitamins, it and its active forms participate in lipid metabolism, which is related to increasing concentrations of total cholesterol, low-density lipoprotein and triglycerides [44,45]. Also, in the past years, the increase in overnutrition stemming from the shift in dietary patterns and reduction of physical activity in China [46,47]. Compared with the traditional southern dietary patterns, modern and northern dietary patterns, which have higher percent energy contributions from intake of carbohydrates, Fe, vitamin A, vitamin C, fibre and Ca, and lower percent energy intakes from fats, can easily cause obesity in children and adolescents aged 7–17 years in China [48,49]. Given the results of the present study, public health strategies on vitamin A supplementation should consider the effect of retinol on Chinese urban children and adolescents.

One major limitation of the present study is that, since it is a cross-sectional study, causality cannot be established. More studies are needed to understand the reasons for the higher level serum concentrations of retinol in overnutrified Chinese urban children and adolescents.

## 5. Conclusions

In summary, the present study documented a higher prevalence of overnutrition in children than adolescents in Chinese urban areas, as well as the fact this was higher en males than females. A higher prevalence of inadequate retinol and lower levels of retinal was observed in children. There was a positive correlation between serum concentrations of retinol and children’s body mass index. Non-overnutrition and children easily show inadequate levels of retinol. In further study, public health strategies should focus on the overnutrified population and vitamin A supplements and should consider the effect of retinol on children and adolescents in Chinese urban areas.
